# The Influence of Salt Anions on Heavy Metal Ion Adsorption on the Example of Nickel

**DOI:** 10.3390/ma11030373

**Published:** 2018-03-03

**Authors:** Mandy Mende, Dana Schwarz, Christine Steinbach, Regine Boldt, Simona Schwarz

**Affiliations:** Leibniz-Institut fuer Polymerforschung Dresden e.V., Hohe Str. 6, 01069 Dresden, Germany; mende@ipfdd.de (M.M.); schwarz-dana@ipfdd.de (D.S.); Steinbach@ipfdd.de (C.S.); Boldt@ipfdd.de (R.B.)

**Keywords:** nickel adsorption, oxyanion adsorption, chitosan, natural polymers, polysaccharides, waste water, adsorption kinetics

## Abstract

The biodegradable polysaccharide chitosan possesses protonated and natural amino groups at medium pH values and has therefore been used as an adsorbing material for nickel salts in water treatment. Nickel is a problematic heavy metal ion which can cause various diseases and disorders in living organisms. Here, we show the influence of oxyanions (e.g., nitrate and sulfate) to the adsorption of nickel ions. Hence, simultaneously we are addressing the increasing global problem of nitrate and sulfate ion pollution in groundwater and surface water. A series of adsorption experiments was carried out in order to determine (i) the adsorption equilibrium, (ii) the adsorption capacity in dependence on the initial nickel ion concentration, and (iii) the influence of the anion presented in solution for the adsorption capacity. Surface morphology of chitosan flakes before and after the adsorption process has been studied with SEM-EDX analysis. The chitosan flakes exhibited promising adsorption capacities of 81.9 mg·g^−1^ and 21.2 mg·g^−1^ for nickel (sulfate) and nickel (nitrate), respectively. The calculated values of Gibbs free energy change ΔG^0^ confirm the higher adsorption of nickel ions in presence of sulfate ions. Hence, higher anion valence leads to a higher adsorption capacity.

## 1. Introduction

In recent years, heavy metal ion pollution in water treatment has become a major issue in research due to the voluminous discharge into the environment by mining activities, industrial, and agricultural effluents [1,2]. Nickel is commonly used in electroplating industry and as fertilizer. Exposure to nickel can cause dermatitis, renal disturbances, hepatitis, insomnia, and allergic sensitization. Inhalation of nickel can cause respiratory cancer [3,4]. According to the world health organization (WHO) guidelines, the maximum value of nickel is 4.1 mg·L^−1^ for industrial effluent and less than 0.1 mg·L^−1^ for drinking water [5].

Furthermore, oxyanions like nitrate and sulfate became an increasing environmental issue as well, due to overfertilization and mining. Nitrate ions stimulate the growth of organisms and algaes and is dangerous for aquatic life [6]. The increasing amount of sulfate ions leads to a decreasing pH-value of the water and therefore to damages and corrosion of cement in sewer and bridges for example [7]. Current physical and chemical processes for heavy metal ion as well as for oxyanion removal such as precipitation, filtration, reduction, and ion-exchange are expensive and inefficient in treating large quantities of water [8,9,10]. Adsorption is an efficient and economical process with increasing concern in research during the last years. Popular adsorber materials are activated carbon [11], zeolites [12], clay [13], activated alumina [14] and silica [15]. It is desirable to develop high-capacity adsorbents that are cheap to fabricate and easy to operate. Biosorption on widely available biopolymers is a feasible alternative to common processes due to their low operating costs and minimization in the usage of chemicals [16,17]. Among the potential low-priced solid adsorbents, chitosan exhibits high adsorption capacities for several heavy metal ions [18,19].

Chitosan is a biodegradable polysaccharide and originates from the natural polymer chitin [20]. Latter is mainly obtained from the shells of crustacean and is therefore the second most abundant polymer in nature. Chitosan is a crystalline copolymer of manly glucosamine, and N-acetyl glucosamine units [21]. The field of applications for chitosan is huge and increases constantly due to its unique sets of characteristics: biodegradability, biocompatibility, antibacterial, hydrophilicity, and nontoxicity [22,23]. Particularly the implementation as an adsorber material in wastewater and drinking water treatment for various kinds of impurities (e.g., heavy metal ions, anions, pharmaceutics, or dyes) has attracted a lot of attention in recent years.

The long known good adsorption of heavy metal ions on chitosan is mainly attributed to the presence of amine groups at the C2 position [24,25]. The amine groups lead to certain properties of the chitosan, such as (i) cationic polyelectrolyte (pKa ≈ 6.5), (ii) solubility in aqueous acetic media, (iii) formation of water insoluble complexes with anionic polyelectrolytes [26], and (iv) chelation of metal ions [27]. Additionally, the hydroxyl group in C3 position of the chitosan unit is available as binding site too [28]. At low pH-value in solution, chitosan acts as a weak basic anion exchange resin due to the protonated amine groups. Metal anions, such as arsenate [29], can interact with the positive charge of the protonated amine groups by electrostatic interaction. With increasing pH-value the overall positive charge on chitosan surface decreases. Hence, the formation of chelate complexes between metal cations and the lone pair of electron on the nitrogen atom of the uncharged amine groups is preferred. A good comprehensive survey about chitosan and their ability to interact with ions is given by Guibal [30].

Previously, we have shown that chitosan offers high adsorption capacities for copper, iron and nickel ions. Among those three ions, copper exhibited the highest adsorption capacity with 150 mg·g^−1^ [31]. Analyses by scanning electron microscopy coupled with energy dispersive X-ray spectroscopy (SEM-EDX) indicated the simultaneous adsorption of the anion presented in metal ion solution and the metal cation. Hence, in a more detailed study the simultaneous adsorption of iron and sulfate ions from aqueous solution was represented by Ultraviolet-visible (UV/Vis) measurements of the solution [32].

The aim of the present study was to investigate the adsorption behavior of nickel on chitosan flakes in dependence on contact time, initial nickel concentration, and the corresponding oxyanions. The valence of the associated anion was considered in the discussion. Adsorption capacities were determined without adjusting the pH of the initial metal salt solutions. In order to understand the adsorption behavior, the experimental adsorption data were fitted to Langmuir, Freundlich, and Langmuir-Freundlich isotherm models. Kinetic parameters were determined by fitting the isotherms to the pseudo first order, pseudo second order, and intra-particle diffusion model. Additionally, selected samples were analyzed by scanning electron microscopy (SEM) and energy dispersive X-ray spectroscopy (EDX) to get more information about the adsorption mechanism.

## 2. Materials and Methods 

### 2.1. Materials

#### 2.1.1. Chitosan

Chitosan flakes with a degree of deacetylation of 90% were purchased from the BioLog Heppe^®^ GmbH, Landsberg, Germany and used as received. The wide particle size distribution of the flakes was in mm range. 

#### 2.1.2. Salts

The used nickel salts (nickel (II) sulfate hexahydrate and nickel (II) nitrate hexahydrate) were purchased from Sigma-Aldrich (Steinheim, Germany) and used as received.

### 2.2. Adsorption Experiments

Adsorption investigations were carried out as batch experiments. The suspensions were stirred with a magnetic stirrer for a desired time at 25 °C. Subsequently, the samples were filtered with a Sartorius 389 filter paper. The nickel ion concentrations were determined by absorbance measurements before and after the adsorption process.

To study the effect of contact time on ion adsorption 20 mL of the nickel salt solution with an initial cation concentration of 180 mg·L^−1^ were mixed with 0.1 g chitosan flakes in a 100 mL beaker. The initial pH-values of nickel sulfate and nickel nitrate solutions were 6.1 ± 0.05. During adsorption experiments, the pH did not change significantly.

Experiments to elucidate the effect of initial metal ion concentration on equilibrium were realized by adding 20 mL of metal salt solutions with different initial salt concentration to 0.1 g chitosan for a contact time of 24 h. Formulas used for the calculations can be found in the Supporting Information (SI).

### 2.3. Analytical Methods

#### 2.3.1. Spectrophotometry

DR2800 of the HACH Lange GmbH, Düsseldorf, Germany, was used to determine the metal cation concentration in solution before and after the adsorption process. It is a visible light spectrophotometer with pre-programmed test methods, so-called cell tests. During the testing process each sample is rotated and 10 measurements were done to give a concentration value (or transmittance or absorbance). The test for nickel ions is based on the formation of an orange brown precipitate with dimethylglyoxime in alkaline solution.

#### 2.3.2. SEM

SEM images were detected with the scanning electron microscope Ultra plus from Carl Zeiss NTS, Oberkochen, Germany. All samples were prepared on a graphite carrier and coated with a 3 mm layer of platinum.

#### 2.3.3. SEM-EDX analysis

The chitosan surfaces were analyzed with the combination of scanning electron microscopy (SEM) and energy dispersive X-ray spectroscopy (EDX) before and after adsorption processes. It was done with the Ultra Plus from Carl Zeiss NTS, Oberkochen, Germany equipped with an EDX-detector from Bruker Nano GmbH, Berlin, Germany. All samples were coated with carbon before the SEM-EDX analysis.

## 3. Results and Discussion

### 3.1. Adsorption Equilibrium

**Figure 1:**
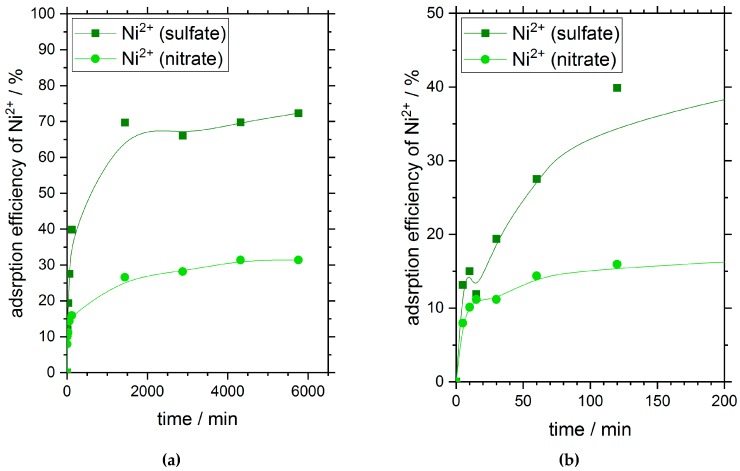
Adsorption efficiency of nickel ions in dependence on time and their associated anions at 25 °C. (**a**) displays the full period of time (6000 min) and indicates the adsorption equilibrium in dependence of time; (**b**) shows the first 200 min of the measurement displayed in (**a**) and gives evidence of a two-stage adsorption process. Initial nickel concentration and initial pH-values of the sulfate and nitrate solution were 180 mg·L^−1^ and 6.1 ± 0.05, respectively.

Figure 1 shows the adsorption efficiency of chitosan flakes for nickel ions in dependence on time and the corresponding oxyanion presented in solution. The initial nickel concentration for both the sulfate salt as well as the nitrate salt was 180 mg·L^−1^. The pH-values of the salt solutions were not adjusted and were nearly the same for both salt solutions with 6.1 ± 0.05. During the adsorption process, no significant pH changes were observed.

After 24 h (1440 min), nickel ions have reached their adsorption equilibrium independent on the type of oxyanion used. The curve progression is similar for both adsorptions. Within the first 10 minutes of the adsorption process, about 10% of ions were adsorbed on the chitosan surface. Subsequently, the adsorption process carries on with a slower speed (see Figure 1b).

Very interesting is the distinct difference in adsorption efficiency of nickel on chitosan flakes at equilibrium state in dependence of the oxyanions with different ion valence presented in solution. When sulfate anions with a valence of two are available in solution the adsorption efficiency of nickel in equilibrium is about 70%. In comparison, the adsorption efficiency of nickel ions with a valence of one for the nitrate anions in solution reached a value of about 30% at equilibrium.

However, the different adsorption efficiencies after reaching the adsorption equilibrium give first evidence of a strong adsorption dependency on the corresponding anions of the used heavy metal salt.

### 3.2. Adsorption Isotherms

**Figure 2:**
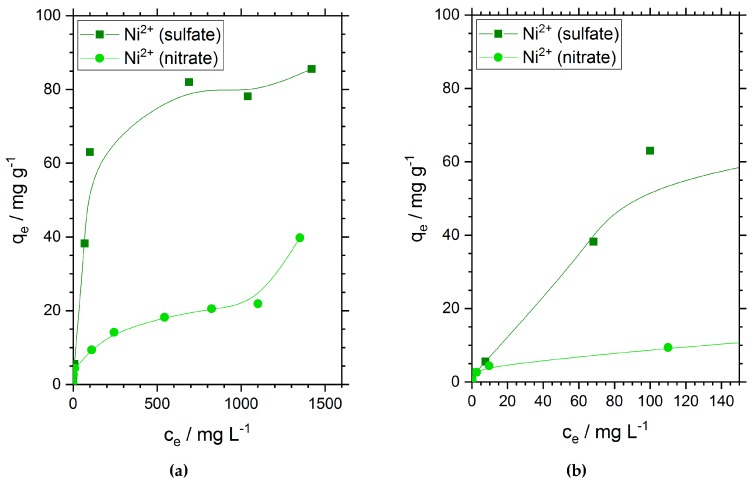
Adsorption isotherms of nickel at 25 °C. (**a**) displays the full measurement and indicates the adsorption equilibrium in dependence of the concentration; (**b**) shows a close up of (**a**) for very low concentrations. Initial pH-values range from 5.8 to 6.3.

The adsorption capacities of chitosan flakes for nickel ions are shown in Figure 2. Similar to the results observed for the adsorption efficiency in Figure 1, we found a much higher adsorption capacity of 81.9 mg·g^−1^ for nickel ions when sulfate ions are presented in solution. This value is three times higher than the adsorption capacity of nickel ions when nitrate is the oxyanion. In the case of nickel nitrate solution, we found an experimental value of 21.2 mg·g^−1^. The obtained adsorption capacities were compared with the adsorption capacities reported in other references for chitosan and other materials in the supporting information (SI). As long as it was mentioned we also stated the type of nickel salt used for the investigation. High adsorption capacities were often obtained for nickel sulfate. In comparison, lower adsorption capacities were in many cases obtained for nickel nitrate and nickel chloride salts. This adsorption dependency on the anion fits well with our results. However, this is just one parameter among many which can all have an impact on the adsorption capacity. The comparison of our results with the literature can be seen in Table S2, SI. In general, the obtained adsorption capacity for nickel sulfate on chitosan is very high in comparison to other materials. 

The initial slope of both curves and the shape of the adsorption isotherms seem to correspond to the IUPAC type I that should be specific for microporous adsorbents with a relatively small outer surface and Giles classification L [33]. According to Giles, the L curve indicates no strong competition between adsorbate and solvent for sites on the adsorbent surface.

**Figure 3:**
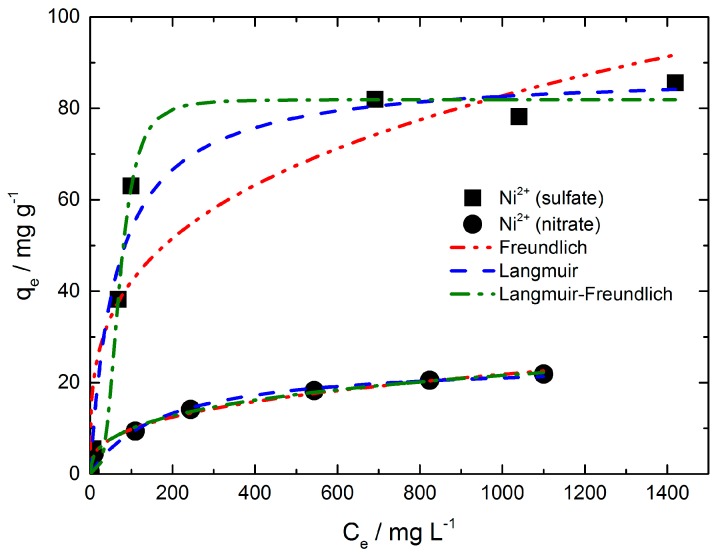
Adsorption isotherm models for nickel adsorption on chitosan flakes from nickel sulfate and nickel nitrate solution. The adsorption time and the initial pH-values were 24 h and 5.8 to 6.3, respectively.

The well-known Langmuir, Freundlich and Langmuir-Freundlich isotherm models were used to analyze the experimental data shown in Figure 3.

The equilibrium parameters derived from the Langmuir, Freundlich and Langmuir-Freundlich models are summarized in Table S1, SI. The Freundlich model gave a good fit for the equilibrium data of nickel adsorption from nickel nitrate solution (R^2^ = 0.995). In contrast, the fit of the equilibrium data for the nickel (sulfate) ion adsorption by the Freundlich model is not sufficient (R^2^ = 0.892). Nevertheless, the Freundlich adsorption coefficient $K_{F}$ indicates that the adsorption strength is distinct higher for nickel when sulfate anions are present in solution. The adsorption strength is directly correlated with the adsorbent loading. The energetic heterogeneity of the adsorbent surface is deduced by the exponent $n_{F}$. The $n_{F}$ values are greater than 2 which means that good adsorption takes place in both cases and the isotherms conform type I ($n_{F}$ > 1).

The Langmuir model provided a better fit for the nickel ion adsorption from sulfate solution (R^2^ = 0.979). In comparison to the Freundlich fit, the fit for the nickel (nitrate) ion adsorption resulted in a less sufficient fit (R^2^ = 0.963). Since both isotherms (i.e., nickel sulfate and nickel nitrate) feature different curve progressions, the correlation coefficient can differ depending on the used model. For each one the model with the best fitting was chosen. The nickel (sulfate) isotherm possesses a steep increase at low concentration values and reaches a saturation range (q = constant) relatively rapidly leading to a strong curvature of the isotherm. In comparison, the nickel (nitrate) isotherm features only a minor curvature with a range over the total isotherm. The Langmuir model is often not suitable due to the criteria of monolayer coverage of the adsorbent and energetic homogeneity of the adsorption sites which must be fulfilled. Hence, the nickel (sulfate) ion adsorption might fulfill the monolayer coverage of the adsorbent better than nickel (nitrate) ions featuring a clearly smaller adsorption capacity in comparison to nickel (sulfate) ions. In general, the Langmuir model is more suitable for isotherms like nickel sulfate (i.e. steep linear increase at low concentration ranges, a strong curvature followed by a saturation range) and the Freundlich model represents better the medium concentration range as observed for the nickel nitrate isotherm. Maximum amounts (q_m_) of adsorbed nickel ion were found to be near the experimentally determined ones shown in Table S1, SI. The separation factor $R_{L}$ ranges between 0 and 1 for the adsorption of nickel ions from both nickel salt solutions indicating favorable isotherms. Though the values of $R_{L}$ were only slightly below 1 for both adsorption isotherms at lower initial nickel ion concentrations. When nickel ions were adsorbed from a nickel sulfate solution the initial nickel concentration of 3.55 mg·L^−1^ gives a $R_{L}$ value of 0.946. With increasing initial nickel concentration, the $R_{L}$ value decreases to 0.033. The same behavior was observed for the adsorption of nickel ions from nickel nitrate solution. Admittedly the $R_{L}$ values were slightly higher at the same initial nickel concentration compared to nickel adsorption from sulfate solution.

Best fitting of the equilibrium data of both nickel adsorption iostherms seems to be given by the Langmuir-Fredlich model with a R^2^ value of 0.991 and 0.996 (see Table S1, SI). In comparison to the Langmuir and Freundlich model, the Langmuir-Freundlich model introduces a third parameter and is therefore not so much restricted. On one hand, the calculated adsorption capacity ($q_{m}$) for nickel sulfate solution corresponds very well with the experimental determined one. On the other hand, there is a distinct difference of the calculated $q_{m}$ for nickel nitrate solution compared to the experimental value.

The heterogeneity parameter $n_{\mathrm{LF}}$ is lower than one for the nickel (nitrate) ion adsorption indicating a heterogeneous surface as it is frequently described in the literature. A value of one would show the adsorption on a homogeneous surface (Langmuir). However, as one can expect the surface of chitosan flakes should be not so uniform. Unusual high, however, is the $n_{\mathrm{LF}}$ value for the nickel sulfate solution with 3.475. Although $n_{\mathrm{LF}}$ should be lower than one according to theory, values above one have been reported in several papers. Among others, Nam et al. observed $n_{\mathrm{LF}}$ > 1 for the adsorption of several gases on zeolites especially for ethen (293.15 K, $n_{\mathrm{LF}}$ = 3.24) and ethan (293.15 K, $n_{\mathrm{LF}}$ = 2.57) in which the adsorption isotherms were of type I [34]. Deshpande et al. reported a $n_{\mathrm{LF}}$ value above one for the adsorption of methyl green on carbon-zirconia composite [35]. Oubagaranadin and Murthy reported values greater and smaller than one for the adsorption of ions on activated clay [36].

However, why is $n_{\mathrm{LF}}$ of the nickel sulfate solution so high in comparison to $n_{\mathrm{LF}}$ of the nickel nitrate solution even though the initial surface of used chitosan flakes should have nearly the same characteristics and other conditions like the initial pH are equal? We know that from nickel sulfate solution much more nickel is adsorbed at the same initial nickel concentration in equilibrium compared to nickel nitrate solution. Apparently, there is no monomolecular coverage of chitosan surface and with the growth of adsorbed layers the surface would become smoother and be more uniform, respectively, regarding the sites available for (further) adsorption. 

### 3.3. Thermodynamic Consideration

Based on the equilibrium data of the isotherms, the Gibbs free energy change (ΔG^0^) was calculated with Equation 7 (see SI). The calculated values of $K_{C}$. and ΔG^0^ are summarized in Table 1. The equilibrium constant decreases with increasing initial nickel concentration for both nitrate and sulfate solutions and very interestingly, the change in Gibbs free energy from negative to positive values with increasing initial nickel concentration in both cases too. It is known that the absolute magnitude of the change in free energy for physisorption is between −20 and 0 kJ·mol^−1^. Chemisorption has a range of −80 to −400 kJ·mol^−1^ [37]. The results show that at a lower initial concentration of nickel ions the adsorption process in both cases is a physisorption. Hence, the adsorption is based on electrostatic interaction. As long as the values of ΔG^0^ are negative the adsorption of nickel is a spontaneous process. At higher initial nickel concentrations, positive values of ΔG^0^ were calculated which indicates that the adsorption decreases until equilibrium has reached. This point, the change from negative to positive ΔG^0^ values, is reached at a lower initial nickel concentration when nitrate is present in solution (between 31.7–157 mg·L^−1^). In the case of nickel sulfate, the spontaneous adsorption takes place up to an initial nickel ion concentration of 761 mg·L^−1^. The results indicate a better adsorption of nickel in sulfate solution too.

### 3.4. Adsorption Kinetics

**Figure 4:**
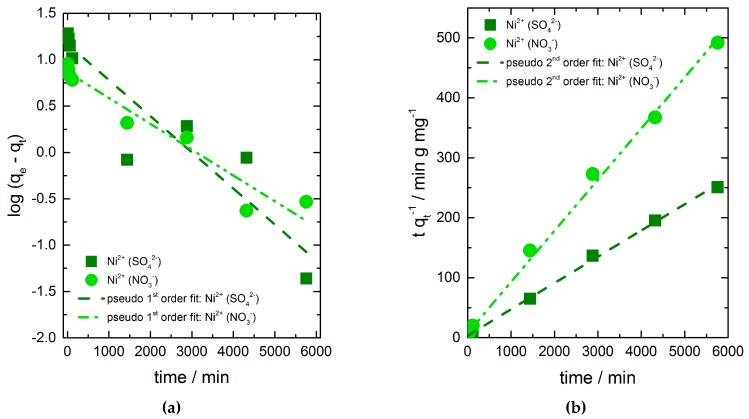
Effect of contact time and the present salt anion on the adsorption of nickel ions on chitosan flakes. Data fits to (**a**) pseudo-first-order and (**b**) pseudo-second-order kinetic model.

To get some information about the rate of the adsorption and the controlling mechanism of the adsorption processes pseudo-first-order, pseudo-second-order and intra-particle diffusion kinetic models were used to fit the data for kinetic parameters (Figure 4a,b). The kinetic parameters obtained from the linearized plots are summarized in Table 2. Best fits are given by the pseudo-second-order kinetic model in both cases, which suggest that the sorption reaction is the rate controlling step of the adsorption process instead of mass transport. The calculated adsorption capacities in equilibrium correspond to the experimental determined values very well.

As pseudo-first-order and pseudo-second-order kinetic models cannot identify the influence of the diffusion mechanism on the adsorption process, the intra-particle diffusion model was applied to the adsorption data too. The adsorption process takes place in four stages: (i) transport in the bulk solution, (ii) diffusion of adsorbate through the liquid film surrounding the adsorbent particle to the surface of the adsorbent, (iii) intra-particle diffusion (diffusion of adsorbate molecules through pores), and (iv) adsorption and desorption of molecules within the particle and on external adsorbent surface [38]. The first and last stages are usually fast and do not control the adsorption process. Transport in solution can also be ignored as the adsorption experiments were done in rapidly stirred batch systems. Hence, the rate limiting step is either the diffusion through the liquid layer or the diffusion through the pores. 

**Figure 5:**
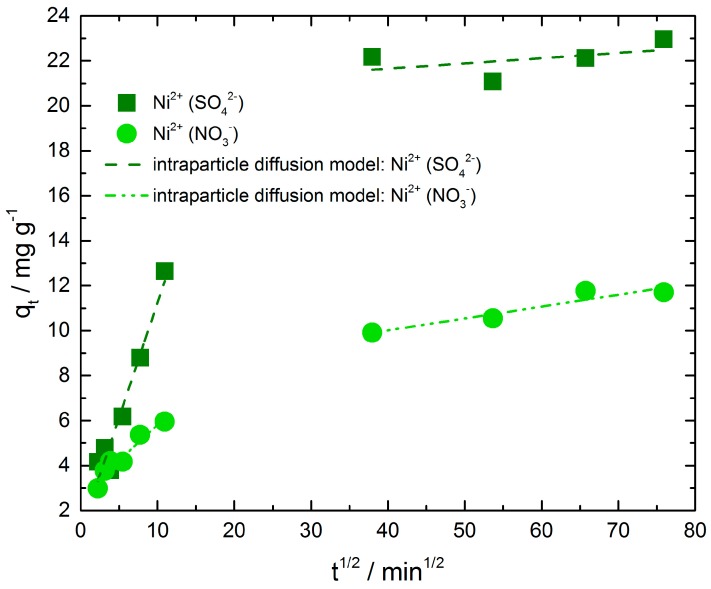
Intra-particle diffusion kinetic plot for nickel adsorption from nickel sulfate and nickel nitrate solution.

Figure 5 presents the intra-particle diffusion plots of nickel ions adsorbed on chitosan from nickel sulfate solution as well as from nickel nitrate solution. The results indicate that the adsorption process of both exhibit two separate steps with two different straight lines. The values of intercept, slope and correlation coefficient (R^2^) were calculated and summarized in Table 3. Deviations of the straight lines from the origin (intercept C, Table 3) indicate that in both adsorption processes the intra-particle diffusion is not the only rate limiting mechanism. Hence, the larger the C value of the intercept, the greater is the boundary layer effect. The second step of nickel adsorption from sulfate solution shows a higher C value compared to the other ones. Obviously, the resistance of boundary layer and therefore their thickness is distinct greater than in step one of the same adsorption and in step one and two of nickel ion adsorption from nitrate solution. It is necessary to point out that the correlation coefficient in this case is very low and hence the intra-particle diffusion model does not describe the mechanism well enough.

Furthermore, the first straight line portions are attributed to macropore diffusion process and the second one suggests diffusion by meso- and micropores of the adsorbent as described in literature [39,40]. In both cases, the first step is faster than the second step. This behavior may be correlated with a very slow diffusion of nickel ions from the surface film into possibly existing micropores. For this reason, chitosan flakes were analyzed by nitrogen adsorption analysis to determine the porosity of the sample as well as the pore sizes (measurements are not presented in this paper). However, the flakes did not show any type of porosity at all. To pursue the idea of very slow diffusion due to possible small micropores CO_2_ adsorption analysis was performed too, because small micropores can be better observed with CO_2_ adsorption analysis. The chitosan flakes did not exhibit any kind of pores in this case too. Gas sorption analysis was carried out with the very carefully dried chitosan flakes. As chitosan is a linear polymer, it might have pores in solution due to a different arrangement of the polymer or swelling depending on the pH and the salt concentration in comparison to the dry polymer. 

A comparison of the intra-particle diffusion rate constants of initial parts reveals a distinct difference between nickel ion adsorption from sulfate and nitrate solution. The rate constant of nickel adsorbed from nickel sulfate solution is higher than the other one. In the second step we observe a slightly higher rate constant for nickel adsorbed from nickel nitrate solution, admittedly the correlation coefficient of the second step of nickel adsorption from nickel sulfate solution is very low as mentioned above and demonstrates that the intra-particle diffusion model does not describe the adsorption mechanism in this region. 

### 3.5. SEM and SEM-EDX Analysis

**Figure 6:**
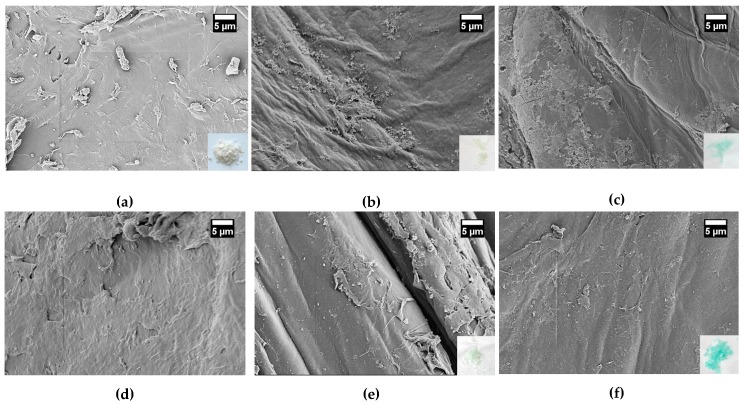
SEM images (magnification 2 kx) of chitosan surfaces (**a**) natural chitosan flake; (**b**) natural chitosan flake after the storage for 24 h in water and followed by air drying; (**c**) and (**d**) after adsorption of nickel ions from nickel nitrate solution with initial nickel ion concentration of (**c**) 35 mg·L^−1^ Ni^2+^ and (**d**) 350 mg·L^−1^ Ni^2+^; (**e**) and (**f**) after adsorption of nickel ions from nickel sulfate solution with initial nickel ion concentration with (**e**) 35 mg·L^−1^ Ni^2+^ and (**f**) 350 mg·L^−1^ Ni^2+^. The contact time of chitosan with nickel solutions was 24 h.

Due to some inconsistencies in interpretation of previous results of nickel adsorption on chitosan in dependence on the oxyanion, the surface of the chitosan flakes was investigated by SEM before and after the adsorption process (contact time 24 h) as shown in Figure 6. Figure 6 a,b display the natural chitosan surface before and after the storage of chitosan flakes in deionized water for 24 h and drying on air afterwards, respectively. Without storage in water natural chitosan flakes show a very uneven and rough surface. Pores were not observed. The surface of chitosan after the storage in water seems to be smoother but not completely uniform. After adsorption of nickel ions on chitosan, the structure of the loaded chitosan surfaces was similar to the chitosan surface stored in water. Regardless some differences were found. 

In Figure 6c,d the chitosan surfaces after nickel ion adsorption from nickel nitrate solution are shown. The initial concentrations of nickel were 35 mg·L^−1^ in Figure 6 c and 350 mg·L^−1^ in Figure 6d. Figure 6e,f present the chitosan surfaces after nickel adsorption from nickel sulfate solution with initial nickel concentration of 35 mg·L^−1^ and 350 mg·L^−1^, respectively. The low insets in the images (bottom right) show chitosan flakes. Natural chitosan flakes are white. After the adsorption of nickel ions, a more or less intense green color of the flakes was observed in dependence on the initial nickel concentration and the oxyanion used. Thus, the absorption of nickel ions is already visually recognized. When the chitosan flakes were treated with a nickel sulfate solution, the color of the chitosan flakes was more intense green after adsorption process compared to the adsorption from a nickel nitrate solution at the same initial nickel concentration. Moreover, the color correlates with the color of the solid nickel salt which was used for nickel adsorption investigations. The increase in color intensity was observed for the adsorption of nickel ions with increasing contact time too (not shown here).

Independent on the oxyanion and the initial nickel ion concentration, an almost uniform coverage of the chitosan surface was observed hence the surface appears smoother than the chitosan surface after storage in water. On this smoothed surface local areas of small crystal-like structures were formed. In previous studies, we observed more clearly grows of crystal-like structures on the chitosan surface with increasing initial concentrations of copper sulfate [41].

For this reason, SEM-EDX analysis was carried out on the chitosan surfaces after nickel ion adsorption from both nickel nitrate and nickel sulfate solution with the initial nickel concentration of 350 mg·L^−1^ and the contact time of 24 h.

**Figure 7:**
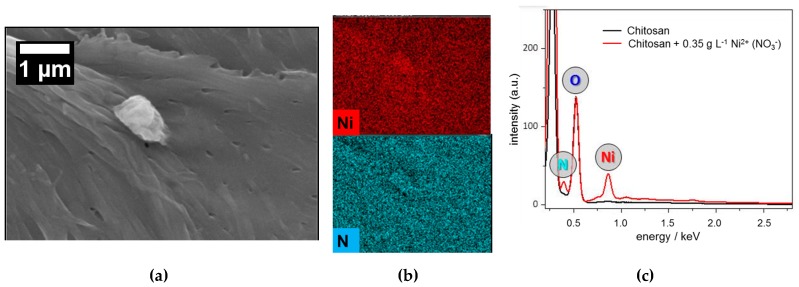

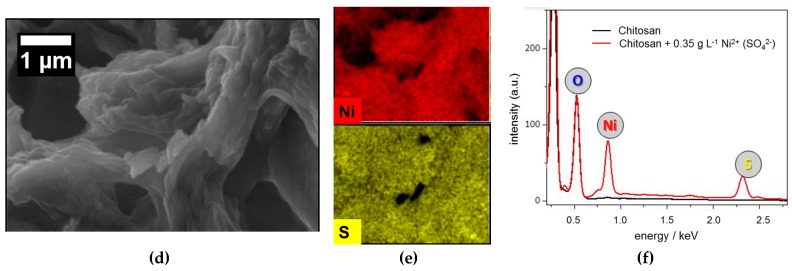
SEM micrographs of the surfaces of chitosan flakes, (**a**) after adsorption of nickel from nickel nitrate solution with (**b**) the corresponding elemental distribution images of nickel and nitrogen and; (**d**) after adsorption of nickel from nickel sulfate solution with (**e**) the corresponding elemental distribution images of nickel and sulfate; (**c**,**f**) show the EDX spectra of natural chitosan surface and chitosan surface after adsorption of nickel from nickel sulfate and nitrate solution, respectively. In both cases initial concentration of nickel was 350 mg·L^−1^ and contact time amounted to 24 h.

In Figure 7 SEM images of chitosan surfaces loaded with nickel from nickel nitrate solution (Figure 7a) and nickel sulfate solution (Figure 7d) are shown with the corresponding distribution images of elements, which were of interest (Figure 7b,e). Furthermore, Figure 7c,f show the EDX spectra received from natural chitosan surface in comparison to the EDX spectra after the adsorption of nickel ions from nickel sulfate solution and nickel nitrate solution, respectively. From the EDX spectrum of natural chitosan (black line) only carbon and oxygen were found overlaying the nitrogen peak. It must be mentioned that all surfaces were covered by carbon before SEM-EDX analysis.

When nickel ion adsorption was investigated with nickel sulfate solution a clear peak was found for sulfur along with the expected nickel peak. Both peaks were not observed in the EDX spectrum of natural chitosan (see Figure 7f). This result indicates that salt cation (nickel) and salt anion (sulfate) adsorb on chitosan surface simultaneously at the pH of about 6 at which the adsorptions were carried out. 

In Figure 7c, the EDX spectrum of natural chitosan is compared to the EDX spectrum of the chitosan after treatment with nickel nitrate solution. The expected nickel peak was observed. The EDX spectra reveal also a slightly higher nitrogen peak compared to the natural chitosan surface, which should come from the additional nitrogen adsorbed by nitrate on chitosan surface.

From the comparison of EDX spectra of nickel ion adsorption from sulfate as well as nitrate solution shown in Figure S1 difference between the nitrogen peaks was found too. The nitrogen peak in the EDX spectrum received from chitosan surface after nickel adsorption from nickel sulfate solution (red line) should arise from the nitrogen contained in chemical structure of chitosan. The slightly higher nitrogen peak in EDX spectrum of the chitosan surface after adsorption of nickel from nickel nitrate solution (blue line) should be the sum of nitrogen contained in the chemical structure of chitosan and the nitrate ion adsorbed on the chitosan surface. EDX spectra in Figure S1 confirmed a higher nickel adsorption on chitosan from nickel sulfate solution too. The nickel peak in the EDX spectrum of chitosan after the adsorption of nickel from nickel sulfate solution is clearly higher than that of nickel adsorbed from nickel nitrate solution. Distribution images of the elements nickel, sulfur and nitrogen reveal a fairly uniform coverage of the chitosan surface according to the irregularities and asperities of the surface.

As mentioned above the adsorption potential of chitosan is mostly postulated due to the chelating behavior of the amine groups that presented on chitosan surface at pH close to neutrality and weak acidity, respectively. The streaming potential-pH profile of the chitosan flakes is displayed in Figure S2. At the investigated pH value, the chitosan flakes exhibit a positive charge. Therefore, the amino groups in the chemical structure of chitosan are partially protonated. Moreover, metal anions, such as arsenate, can interact with the positive charge of protonated amine groups by electrostatic interaction and the adsorption of strontium(II) and barium(II)ions on chitosan occurs in the presence of carbonate due to the formation of ternary complexes [42].

Deduced from our results we assume that oxyanions adsorb on the chitosan surface beside cations due to (i) the protonated amine groups, which occur on the chitosan surface beside the uncharged amine groups at the pH of about 6 we worked, and (ii) the positively charged heavy metal ions partially already bonded to the chitosan surface. 

**Figure 8:**
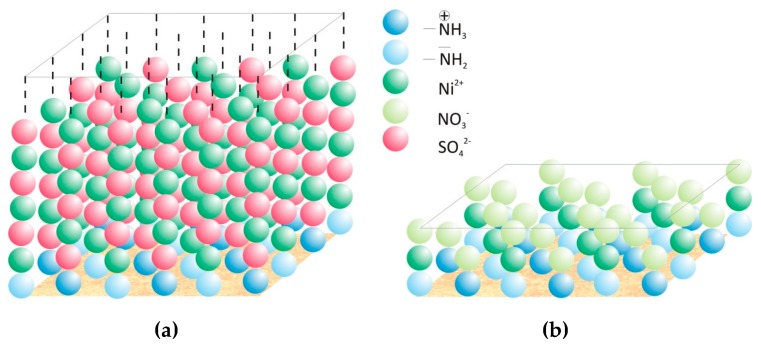
Schematic depiction of the adsorption mechanisms of nickel cations on chitosan surface. Adsorption of nickel from (**a**) nickel sulfate solution and (**b**) nickel nitrate solution.

Figure 8 shows schematically the adsorption process of nickel ions on the chitosan surface from nickel sulfate solution (Figure 8a) and nickel nitrate solution (Figure 8b), which explain the influence of the valence of the oxyanion on the adsorption capacity of chitosan for nickel. Caused by the higher valence of sulfate ions and thereby the possibility of further electrostatic interaction even after a monomolecular covering of some parts on the chitosan surface (deacetylation degree of 90%) the growing of several adsorption layers is given. Further electrostatic interactions of ions in solution with already bounded ions should at least be limited in the case of nitrate ions due to the lower valence of one. Hence, the adsorption capacity of nickel is distinct higher when sulfate ions were presented in the metal ion solution.

## 4. Conclusions

Chitosan with a degree of deacetylation of 90% is a good adsorbent for heavy metal ions in water. The dependence of the adsorption capacity of chitosan flakes for nickel ions was investigated on adsorption time, initial nickel concentration and the valence of the oxyanion (i.e., sulfate and nitrate). In principle, with increasing the initial metal salt concentration the adsorption capacity increases. The equilibrium data were analyzed with Langmuir, Freundlich, and Langmuir-Freundlich isotherm models. The correlation coefficient R^2^ reveals that Langmuir-Freundlich gave the best fit for both nickel salts in solution suggesting heterogeneous surface adsorption of nickel on chitosan flakes. 

The pseudo-first-order, pseudo-second-order and intra-particle diffusion models were used to describe the kinetic data and for determining and evaluate the rate constants. The experimental data of nickel adsorption from sulfate as well as nitrate ion solutions fitted well to the pseudo-second-order-model, which indicates that sorption is the rate-limiting step, instead of mass transfer.

Additionally, the calculation of the Gibbs free energy change ΔG^0^ revealed that the adsorption of nickel ions on chitosan is a spontaneous process up to a certain initial concentration, which is higher in the case of sulfate solution.

On chitosan surface a simultaneous adsorption of cations and anions of the used salt occurs in a medium pH range, where both protonated and non-protonated amino groups are available on the surface of chitosan revealed by SEM-EDX analysis. The elements are fairly uniform distributed over the whole chitosan surface. 

Caused by the different valences of the oxyanions a distinct difference of the adsorption capacity of chitosan for nickel ions is observed. Higher anion valence leads to a higher adsorption capacity due to the possibility of more electrostatic interactions between the cations and anions in solution and already adsorbed ions.

Future investigations should include the exact determination of the structures that are formed on the surface of chitosan during adsorption. Furthermore, it is important for a practical application to determine the extent to which the adsorption of the two types of ions (i.e., cation and anion) on the chitosan surface can be influenced by the pH value and the effects on the adsorption capacities of both ion types. Hence, it is possible to affect the selectivity of the adsorption by pH changes.

## Figures and Tables

**Table 1:** Equilibrium constant K_C_ and Gibbs free energy change ΔG^0^ at T = 298 K of the adsorption of nickel on chitosan in dependence on the initial concentration and the salt anion in solution. The contact time was 24 h.

Ni(NO_3_)_2_				NiSO_4_			
C_0_	C_e_	K_C_	ΔG^0^	C_0_	C_e_	K_C_	ΔG^0^
(mg·L^−1^)	(mg·L^−1^)		(kJ·mol^−1^)	(mg·L^−1^)	(mg·L^−1^)		(kJ·mol^−1^)
1550	1350	0.15	4.73	1850	1420	0.30	2.96
1210	1100	0.10	5.70	1430	1040	0.38	2.43
927	824	0.13	5.15	1100	691	0.59	1.30
636	544	0.17	4.40	761	180	3.23	−2.90
315	244	0.29	3.06	417	100	3.17	−2.86
157	110	0.43	2.11	260	68.2	2.81	−2.56
31.7	9.63	2.29	−2.05	35.5	7.59	3.68	−3.23
15.8	2.64	4.98	−3.98	15.2	2.08	6.31	−4.56
3.15	0.166	17.98	−7.16	3.55	0.095	36.37	−8.90

**Table 2:** Kinetic parameters determined from pseudo-first-order and pseudo-second-order model of nickel adsorption on chitosan in dependence on salt anion presented in solution.

Ni^2+^ Salt	Pseudo First Order Model			Pseudo Second Order Model			q_e,exp_ (mg·g^−1^)
	k_1_ (min^−1^)	q_e,cal_ (mg·g^−1^)	R^2^	k_2_ (g·mg^−1^·min^−1^)	q_e,cal_ (mg·g^−1^)	R^2^	
sulfate	8.97×10^−4^	14.81	0.861	5.543×10^−4^	22.82	0.999	22.08
nitrate	6.41×10^−4^	7.34	0.941	9.735×10^−4^	11.72	0.997	11.73

**Table 3:** Kinetic parameters from intra-particle diffusion model for the adsorption of nickel from nickel sulfate and nickel nitrate solutions.

Ni^2+^ Salt	Sulfate	Nitrate
k_i1_ (mg·g^−1^·min^−1/2^)	1.020	0.318
C_i1_ (mg·g^−1^)	1.036	2.634
R^2^	0.952	0.931
k_i2_ (mg·g^−1^·min^−1/2^)	0.023	0.053
C_i2_ (mg·g^−1^)	20.726	7.896
R^2^	0.243	0.907

## Supplementary Materials

The following are available online at http://www.mdpi.com/1996-1944/11/3/373/s1, Equations for the calculations; Table S1: Experimentally determined adsorption capacities and equilibrium parameters; Table S2: Comparison of the obtained nickel adsorption capacity values with the literature; Figure S1: comparison of EDX spectra of pure chitosan and chitosan after treatment with nickel sulfate and nickel nitrate respectively; Figure S2: Streaming potential-pH profile of the chitosan fakes.
