# Supplementary material for: The Influence of Salt Anions on Heavy Metal Ion Adsorption on the Example of Nickel
Source: Materials (Basel). 2018 Mar 3;11(3):373. doi: 10.3390/ma11030373 (PMC5872952; doi:10.3390/ma11030373)
Supplement: Supplementary file 1 [file materials-11-00373-s001.pdf]

Supplementary Materials: The Influence of Salt Anions on Heavy Metal Ion Adsorption on the Example of Nickel

Mandy Mende [1], Dana Schwarz [1], Christine Steinbach [1], Regine Boldt [1] and Simona Schwarz [1,*]

Table of contents

Formulas and Fittings of the isotherms..2
Experimentally determined adsorption capacities and equilibrium parameters 5
Comparison EDX spectra 6
Streaming potential – pH measurements...7
Adsorption capacity of nickel...8
References...10

Formulas and Fittings of the isotherms

The adsorption efficiency in percentage of nickel was calculated according to the following equation:

$$\text{adsorption efficiency in \%} = \frac{(C_0\text{-}C_f)}{C_0} \cdot 100 \quad (1)$$

where C_0 and C_f are the initial and final nickel concentration in mg L^{-1}, respectively.

The amount of nickel adsorbed on the surface of chitosan flakes at equilibrium Q_e was calculated using the mass balance equation:

$$q_e = \frac{(C_0\text{-}C_e)\cdot V}{m} \quad (2)$$

where C_0 and C_e (mg L^{-1}) are the initial and equilibrium concentrations of nickel ions, respectively. V (L) is the volume of the solution with the adsorbate and m (g) is the mass of the adsorbent.

Langmuir isotherm model is based on several assumptions that include the following:

1. a homogeneous surface,
2. adsorption takes place at a fixed number of definite localized sites,
3. all sites are equivalent and have the same energy,
4. at each site only one molecule is able to adsorb and
5. no interaction between adsorbed molecules takes place.

When the surface is saturated by a monomolecular layer of adsorbate the maximum adsorption is reached. The Langmuir isotherm is given by the following equation[1]:

$$q_e = \frac{q_m K_L C_e}{1+K_L C_e} \quad (3)$$

where C_e (mg L^{-1}) is the equilibrium concentration of nickel, q_e (mg g^{-1}) is the equilibrium nickel concentration in the solid phase, q_m (mg g^{-1}) is the maximum amount of adsorption and K_L (L mg^{-1}) is the Langmuir adsorption equilibrium constant. The favorability of the adsorption is given by the dimensionless separation factor R_L which is given by equation (4)[2–4]:

$$R_L = \frac{1}{1+K_L C_0} \quad (4)$$

where C_0 (mg L^{-1}) is the initial nickel concentration and K_L is the Langmuir constant. R_L indicates whether the isotherm is unfavorable ($R_L > 1$), linear ($R_L = 1$), favorable ($0 < R_L < 1$) or irreversible ($R_L = 0$).

Freundlich adsorption isotherm model is a relationship between the amounts of ions (nickel) adsorbed per unit mass on adsorbent (q_e) and the concentration of the ions (nickel) at equilibrium (C_e). It is an empirical relationship describing the adsorption of solutes from a liquid to a solid surface with the assumption that different sites with several adsorption energies are involved (heterogeneous surface), which lead to multilayer adsorption. Interactions between adsorbed molecules are considered. The more molecules are absorbed, the more difficult is the sorption of additional molecules. The Freundlich model is represented by equation (5)[5]:

$$q_e=K_F C_e^{1/n_F} \quad (5)$$

where q_e (mg g^{-1}) and C_e (mg L^{-1}) have the same destination as in Langmuir equation, K_F ($mg^{1-1/n}L^{1/n}g^{-1}$) is the Freundlich constant and n_F is a dimensionless constant. K_F is related to the bonding energy and represents the adsorption capacity. A larger K_F means higher adsorption capacity of the adsorbent. The constant n_F depicts the adsorption intensity and the degree to which an adsorption process is favorable, respectively. A value of n_F less than 1 means poor adsorption. When n_F is between 1 and 2 a moderately difficult adsorption takes place and a value of n_F between 2 to 10 good adsorption should be observed.[6]

Langmuir-Freundlich isotherm model is also known as sips model and was selected for further analysis. It is essentially a Freundlich isotherm, which approaches at high concentrations a maximum value. Langmuir-Freundlich isotherm is given by equation (6)[7–9]:

$$q_e=\frac{Q_m(K_{LF}C_e)^{n_{LF}}}{1+(K_{LF}C_e)^{n_{LF}}} \quad (6)$$

where K_{LF} (L mg^{-1}) is the Langmuir-Freundlich constant and n_{LF} is the dimensionless Langmuir-Freundlich heterogeneity constant that ranges from 0 to 1 as frequently described in literature. The Langmuir-Freundlich equation trends to the Langmuir isotherm, when the heterogeneity parameter n_{LF} is set to unity and at very low concentrations reduces to the Freundlich isotherm.

The Gibbs free energy change (ΔG^0) was calculated by the following equation[10]:

$$\Delta G^0 = -RT\ln(K_C) \quad (7)$$

where R is the universal gas constant with 8.314 J mol^{-1} K^{-1}, T is the temperature (K) and K_C the equilibrium distribution constant. The constant K_C can be calculated by

$$K_C = C_S/C_e \quad (8)$$

where C_e is the equilibrium concentration (mg L^{-1}) and C_S is the amount sorbed on solid at equilibrium.

The Pseudo-first-order rate equation is given as[1]:

$$\log(q_e-q_t)=\log(Q_e)-\left(\frac{k_1}{2.303}\right)t \quad (9)$$

where q_e is the maximum adsorption capacity (mg g^{-1}), q_t is the adsorption capacity at time t (mg g^{-1}) and k_1 (min^{-1}) is the pseudo-first-order rate constant of the adsorption. The rate constant k_1 and q_e were calculated from the slope ($-\frac{k_1}{2,303}$) and intercept ($\log q_e$) of the linear plots of $\log(q_e-q_t)$ versus t.

Pseudo-second-order equation is given by the following equation[11]:

$$\frac{t}{q_t}=\frac{1}{k_2 q_e^2}+\left(\frac{1}{q}\right)t \quad (10)$$

where q_e (mg g^{-1}) and q_t (mg g^{-1}) have the same destination as in pseudo-first-order equation. k_2 (g mg^{-1} min^{-1}) is the pseudo-second-order rate constant of the adsorption. By the plot of t/q_t versus t, which should give a straight line when pseudo-second-order kinetics are applicable, q_e and k_2 can be determined from slope ($\frac{1}{q_e}$) and intercept ($\frac{1}{k_2 q_e^2}$), respectively.

Intra-particle diffusion model is given by the following equation[12]:

$$q_t = k_i t^{1/2} + C \quad (11)$$

where q_t (mg g^{-1}) is the amount adsorbed at time t (min), k_i (mg g^{-1} min$^{-1/2}$) is the intra-particle diffusion rate constant and C (mg g^{-1}) is the intra-particle diffusion constant and related to the thickness or resistance of the boundary layer in the adsorption process. If intra-particle diffusion was involved in adsorption process, the linear plot of q_t vs $t^{1/2}$ give a straight line and if rate-limiting step was only intra-particle diffusion, the line will pass through the origin.[11]

Experimentally determined adsorption capacities and equilibrium parameters

Table S1. Experimentally determined adsorption capacities and equilibrium parameters given by the Langmuir, Freundlich and Langmuir-Freundlich models of the adsorption of nickel on chitosan in presence of sulfate or nitrate anions.

		Langmuir				Freundlich			Langmuir-Freundlich			
	$q_{m,exp}$	q_m	K_L	R_L	R^2	K_F	n_F	R^2	q_m	K_{LF}	n_{LF}	R^2
	mg g^{-1}	mg g^{-1}	L mg^{-1}			$mg^{1-1/n}L^{1/n}g^{-1}$			mg g^{-1}	L mg^{-1}		
Ni^{2+} (SO_4^{2-})	81.9	87.90	0.016	0.033-0.946	0.979	10.894	3.41	0.892	81.92	$1.41*10^{-2}$	3.475	0.991
Ni^{2+} (NO_3^-)	21.2	24.60	0.006	0.097-0.981	0.963	1.9735	2.876	0.995	74.34	$1.21*10^{-4}$	0.422	0.996

Comparison EDX spectra

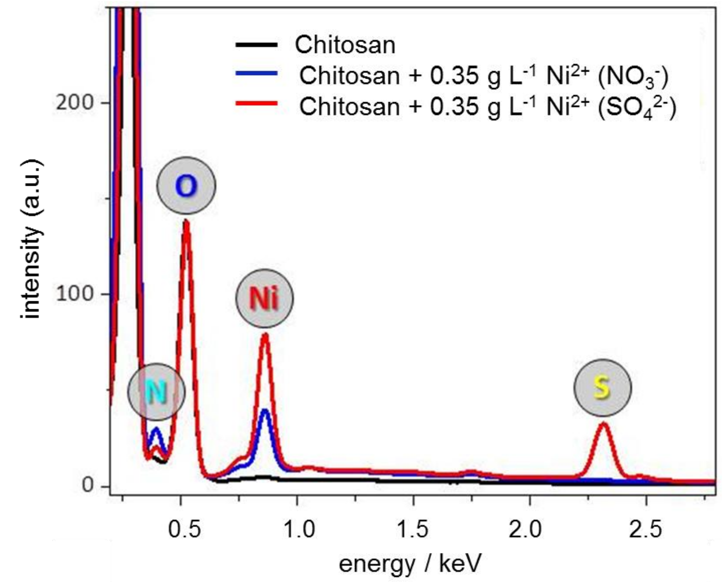

Figure S1. Comparison of the EDX spectra of natural chitosan surface (black line) with chitosan surfaces loaded with nickel sulfate (red line) and nickel nitrate (blue line).

Streaming potential – pH measurements

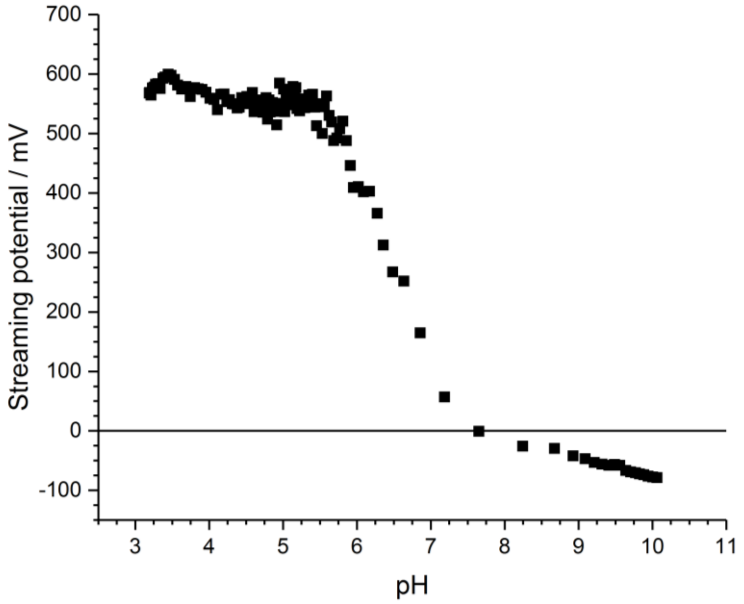

Figure S2. Streaming potential-pH-profile of the chitosan flakes.

Adsorption capacity of nickel

Table S2. Adsorption capacities for nickel (a.d. = adsorbent dose; [a]N,N`-di(carboxymethyl)dithiocarbamate, [b]oxidized multiwall carbon nanotubes; [c]silica gel modified by triethylenetatraaminomethylenephosphonic acid).

Material	Adsorption capacity, q	Ni^{2+} salt	Experimental conditions			Ref.
			pH_0	a.d. (g/L)		
Activated red mud	160 mg/g	$NiSO_4$	5.0	n.a.	20-25°C 600 s	[13]
Activated carbon from apricot waste	101.01 mg/g	$NiNO_3$	5	70	1 h	[14]
Chitosan flakes 90% da Biolog®	81.9 mg/g 1.39 mmol/g	$NiSO_4$	6.0	5.0	25 °C, 24 h	current study
Chitosan/ceramic alumina	78.10 mg/g	$NiSO_4$	4.0	n.a.	25 °C 24 h	[15]
Activated carbon from coirpit	62.5 mg/g	$NiSO_4$	5.0	0.4	30 °C 40 min	[16]
PSDC[a]	0.97 mmol/g	n.a.	5.0	1	288 K, 303 K, 318 K, 24 h	[17]
Blast furnace slag	0.95 mmol/g 55.75 mg/g	$NiCl_2$	4.20	1.0	25 °C 240 min	[18]
Parthenium	54.35 mg/g	$NiSO_4$	5.0	1	12 h 20°C	[19]
Activated carbon from peanut hull	53.65 mg/g	$NiSO_4$	6.5	0.85	24 h	[20]
Chitosan/magnetite	52.55 mg/g	$NiSO_4$	4-6	0.1	120 min RT	[21]
Activated carbon from almond husk	30.769 mg/g	$NiSO_4$	5.0	5	20 °C 10 min	[22]
PEI-Silica gel	28.25 mg/g	$NiNO_3$	5.5	3.0	2 h	[23]
Peat	28.26 mg/g	n.a.	4.5	4.0	25 °C 3 h	[24]
Blank alginate beads	25.6 mg/g	NiNO3	5.0	2.5	25 °C	[25]
Na-bentonite	24.2 mg/g	n.a.	6.0	2.5	22°C 6 h	[26]
Chitosan flakes 90% da Biolog®	21.2 mg/g 0.36 mmol/g	$NiNO_3$	6.0	5.0	25 °C, 24 h	current study
Chitosan/cellulose	13.21 mg/g	n.a.	5.30	2	12 h 25 °C	[27]
Clinopttilolite rock (Ukraine)	13.03 mg/g	$NiNO_3$	6.2	60	24 h	[28]
Baker`s yeast	11.4 mg/g	$NiSO_4$	6.75	1	24 h	[29]
Grape stalks waste	0.1818 mmol/g	$NiCl_2$	6.0	10	25 °C	[30]
MWCNT[b]	9.43 mg/g	$NiNO_3$	6.55	0.75	303 K	[31]

Chitosan/cotton fibers	7.63 mg/g	n.a.	6.5	4	25 °C	[32]
Begasse fly ash	6.49 mg/g	$NiCl_2$	6.0	10	30 °C 5 h	[33]
Ca-bentonite	6.32 mg/g	n.a.	6.0	10	22°C 6 h	[26]
Modified activated carbon	5.81 mg/g	$NiSO_4$	5.0	50	25 °C 200 min	[34]
GH-T-Pd[c]	0.0964 mmol/g	n.a.	5	2	25 °C 24 h	[35]
Chabazite	4.50 mg/g	n.a.	3 - 6	5	24 h	[36]
bentonite	0.0736 mmol/g	$NiNO_3$	3.0	25	60 min 303 K	[37]
Chitosan/alginate beads	2.40 mg/g	n.a.	5.0	0.05	24 h, 25 °C	[38]
Raw Kaolinite	1.669 mg/g	$NiSO_4$	n.a.	n.a.	298 K 2 h	[39]
Fly ash (seyitomer)	1.160 mg/g	$NiNO_3$	8.0	25 mg/l	20 °C 120 min	[41]
Bagasse fly ash	1.12 mg/g	$NiNO_3$	6.5	10	30°C 80 min	[40]
Fly ash (Afsin-elbistan)	0.987 mg/g	$NiNO_3$	8.0	25 mg/l	20 °C 120 min	[41]
Clinoptilolite	0.48 mg/g	$NiCl_2$	5-7	10 - 40	1 mL / min.	[42]
Silica gel	0.0076 mmol/g	n.a.	4.0-5.45	10	25 °C 4 h	[43]

References

1. Lagergren, S., About the theory of so-called adsorption of soluble substances. *K. Sven. Vetenskapsakad. Handlingar* **1898,** 24, (4), 1-39.
2. Hall, K. R.; Eagleton, L. C.; Acrivos, A.; Vermeulen, T., Pore- and Solid-Diffusion Kinetics in Fixed-Bed Adsorption under Constant-Pattern Conditions. *Industrial & Engineering Chemistry Fundamentals* **1966,** 5, (2), 212-223.
3. Ibrahim Ashour, H. A., F. Sawalha and Munjed Maraqah, A Comparative Study for the Treatment of Refinery Synthetic Wastewater Containing Phenol. *Science, Technology and Development* **2015,** 34, (1), 16-26.
4. García, E.; Medina, R.; Lozano, M.; Hernández Pérez, I.; Valero, M.; Franco, A., Adsorption of Azo-Dye Orange II from Aqueous Solutions Using a Metal-Organic Framework Material: Iron-Benzenetricarboxylate. *Materials* **2014,** 7, (12), 8037.
5. Freundlich, H. M. F., Over the Adsorption in Solution. *The Journal of Physical Chemistry* **1906,** 57, 385-471.
6. Chiou, M.-S.; Li, H.-Y., Equilibrium and kinetic modeling of adsorption of reactive dye on cross-linked chitosan beads. *Journal of Hazardous Materials* **2002,** 93, (2), 233-248.
7. Rajic, N.; Stojakovic, D.; Jovanovic, M.; Logar, N. Z.; Mazaj, M.; Kaucic, V., Removal of nickel(II) ions from aqueous solutions using the natural clinoptilolite and preparation of nano-NiO on the exhausted clinoptilolite. *Applied Surface Science* **2010,** 257, (5), 1524-1532.
8. Ho, Y. S.; Porter, J. F.; McKay, G., Equilibrium Isotherm Studies for the Sorption of Divalent Metal Ions onto Peat: Copper, Nickel and Lead Single Component Systems. *Water, Air, and Soil Pollution* **2002,** 141, (1), 1-33.
9. Miah, M. Y.; Volchek, K.; Kuang, W.; Tezel, F. H., Kinetic and equilibrium studies of cesium adsorption on ceiling tiles from aqueous solutions. *Journal of Hazardous Materials* **2010,** 183, (1), 712-717.
10. Ness, J. M. S. a. H. C. V., *Introduction to Chemical Engineering Thermodynamics*. New York: McGraw-Hill, 1987; Vol. 4th ed.
11. Ho, Y. S.; Ng, J. C. Y.; McKay, G., KINETICS OF POLLUTANT SORPTION BY BIOSORBENTS: REVIEW. *Separation and Purification Methods* **2000,** 29, (2), 189-232.
12. Walter J. Weber, J. C. M., Kinetics of Adsorption on Carbon from Solution. *Journal of the Sanitary Engineering Division* **1963,** 89, (2), 31-60.
13. Zouboulis, A. I.; Kydros, K. A., Use of red mud for toxic metals removal: The case of nickel. *Journal of Chemical Technology & Biotechnology* **1993,** 58, (1), 95-101.
14. Erdoğan, S.; Önal, Y.; Akmil-Başar, C.; Bilmez-Erdemoğlu, S.; Sarıcı-Özdemir, Ç.; Köseoğlu, E.; İçduygu, G., Optimization of nickel adsorption from aqueous solution by using activated carbon prepared from waste apricot by chemical activation. *Applied Surface Science* **2005,** 252, (5), 1324-1331.
15. Boddu, V. M.; Abburi, K.; Randolph, A. J.; Smith, E. D., Removal of Copper (II) and Nickel (II) Ions from Aqueous Solutions by a Composite Chitosan Biosorbent. *Separation Science and Technology* **2008,** 43, (6), 1365-1381.
16. Kadirvelu, K.; Thamaraiselvi, K.; Namasivayam, C., Adsorption of nickel(II) from aqueous solution onto activated carbon prepared from coirpith. *Separation and Purification Technology* **2001,** 24, (3), 497-505.
17. Jing, X.; Liu, F.; Yang, X.; Ling, P.; Li, L.; Long, C.; Li, A., Adsorption performances and mechanisms of the newly synthesized N,N′-di (carboxymethyl) dithiocarbamate chelating resin toward divalent heavy metal ions from aqueous media. *Journal of Hazardous Materials* **2009,** 167, (1), 589-596.
18. Dimitrova, S. V., Metal sorption on blast-furnace slag. *Water Research* **1996,** 30, (1), 228-232.
19. Kadirvelu, K.; Senthilkumar, P.; Thamaraiselvi, K.; Subburam, V., Activated carbon prepared from biomass as adsorbent: elimination of Ni(II) from aqueous solution. *Bioresource Technology* **2002,** 81, (1), 87-90.
20. Periasamy, K.; Namasivayam, C., Removal of nickel(II) from aqueous solution and nickel plating industry wastewater using an agricultural waste: Peanut hulls. *Waste Management* **1995,** 15, (1), 63-68.
21. Tran, H. V.; Tran, L. D.; Nguyen, T. N., Preparation of chitosan/magnetite composite beads and their application for removal of Pb(II) and Ni(II) from aqueous solution. *Materials Science and Engineering: C* **2010,** 30, (2), 304-310.
22. Hasar, H., Adsorption of nickel(II) from aqueous solution onto activated carbon prepared from almond husk. *Journal of Hazardous Materials* **2003,** 97, (1), 49-57.
23. Ghoul, M.; Bacquet, M.; Morcellet, M., Uptake of heavy metals from synthetic aqueous solutions using modified PEI—silica gels. *Water Research* **2003,** 37, (4), 729-734.

24. Chen, B.; Hui, C. W.; McKay, G., Film-Pore Diffusion Modeling for the Sorption of Metal Ions from Aqueous Effluents onto Peat. *Water Research* **2001,** 35, (14), 3345-3356.
25. Al-Rub, F. A. A.; El-Naas, M. H.; Benyahia, F.; Ashour, I., Biosorption of nickel on blank alginate beads, free and immobilized algal cells. *Process Biochemistry* **2004,** 39, (11), 1767-1773.
26. REMOVAL OF HEAVY METALS FROM WASTE WATERS BY NATURAL AND Na-EXCHANGED BENTONITES. *Clays and Clay Minerals* **2003,** 51, (5), 475-480.
27. Sun, X.; Peng, B.; Ji, Y.; Chen, J.; Li, D., Chitosan(chitin)/cellulose composite biosorbents prepared using ionic liquid for heavy metal ions adsorption. *AIChE Journal* **2009,** 55, (8), 2062-2069.
28. Sprynskyy, M.; Buszewski, B.; Terzyk, A. P.; Namieśnik, J., Study of the selection mechanism of heavy metal (Pb2+, Cu2+, Ni2+, and Cd2+) adsorption on clinoptilolite. *Journal of Colloid and Interface Science* **2006,** 304, (1), 21-28.
29. Padmavathy, V.; Vasudevan, P.; Dhingra, S. C., Biosorption of nickel(II) ions on Baker's yeast. *Process Biochemistry* **2003,** 38, (10), 1389-1395.
30. Villaescusa, I.; Fiol, N.; Martínez, M. a.; Miralles, N.; Poch, J.; Serarols, J., Removal of copper and nickel ions from aqueous solutions by grape stalks wastes. *Water Research* **2004,** 38, (4), 992-1002.
31. Chen, C.; Wang, X., Adsorption of Ni(II) from Aqueous Solution Using Oxidized Multiwall Carbon Nanotubes. *Industrial & Engineering Chemistry Research* **2006,** 45, (26), 9144-9149.
32. Zhang, G.; Qu, R.; Sun, C.; Ji, C.; Chen, H.; Wang, C.; Niu, Y., Adsorption for metal ions of chitosan coated cotton fiber. *Journal of Applied Polymer Science* **2008,** 110, (4), 2321-2327.
33. Srivastava, V. C.; Mall, I. D.; Mishra, I. M., Equilibrium modelling of single and binary adsorption of cadmium and nickel onto bagasse fly ash. *Chemical Engineering Journal* **2006,** 117, (1), 79-91.
34. Satapathy, D. N., G.S., Potassium bromate modification of the granular activated carbon and its effect on nickel adsorption. *Adsorption* **2006,** 12, (2), 147-154.
35. Tian, Y.; Yin, P.; Qu, R.; Wang, C.; Zheng, H.; Yu, Z., Removal of transition metal ions from aqueous solutions by adsorption using a novel hybrid material silica gel chemically modified by triethylenetetraminomethylenephosphonic acid. *Chemical Engineering Journal* **2010,** 162, (2), 573-579.
36. Ouki, S. K.; Kavannagh, M., Performance of Natural Zeolites for the Treatment of Mixed Metal-Contaminated Effluents. *Waste Management & Research* **1997,** 15, (4), 383-394.
37. Donat, R.; Akdogan, A.; Erdem, E.; Cetisli, H., Thermodynamics of Pb2+ and Ni2+ adsorption onto natural bentonite from aqueous solutions. *Journal of Colloid and Interface Science* **2005,** 286, (1), 43-52.
38. Huang, C.; Chung, Y.-C.; Liou, M.-R., Adsorption of Cu(II) and Ni(II) by pelletized biopolymer. *Journal of Hazardous aterials* **1996,** 45, (2), 265-277.
39. Yavuz, Ö.; Altunkaynak, Y.; Güzel, F., Removal of copper, nickel, cobalt and manganese from aqueous solution by kaolinite. *Water Research* **2003,** 37, (4), 948-952.
40. Gupta, V. K.; Jain, C. K.; Ali, I.; Sharma, M.; Saini, V. K., Removal of cadmium and nickel from wastewater using bagasse fly ash—a sugar industry waste. *Water Research* **2003,** 37, (16), 4038-4044.
41. Bayat, B., Comparative study of adsorption properties of Turkish fly ashes: I. The case of nickel(II), copper(II) and zinc(II). *Journal of Hazardous Materials* **2002,** 95, (3), 251-273.
42. Zamzow, M. J.; Eichbaum, B. R.; Sandgren, K. R.; Shanks, D. E., Removal of Heavy Metals and Other Cations from Wastewater Using Zeolites. *Separation Science and Technology* **1990,** 25, (13-15), 1555-1569.
43. Tran, H. H.; Roddick, F. A.; O'Donnell, J. A., Comparison of chromatography and desiccant silica gels for the adsorption of metal ions—I. adsorption and kinetics. *Water Research* **1999,** 33, (13), 2992-3000.
